# Effect of Mouth Rinsing and Antiseptic Solutions on Periodontitis Bacteria in an In Vitro Oral Human Biofilm Model

**DOI:** 10.3390/dj13070324

**Published:** 2025-07-16

**Authors:** Jan Tinson Strenge, Ralf Smeets, Maria Geffken, Thomas Beikler, Ewa Klara Stuermer

**Affiliations:** 1 Department of Oral and Maxillofacial Surgery, University Medical Center Hamburg-Eppendorf, 20246 Hamburg, Germany; j.strenge@uke.de; 2 Department of Oral and Maxillofacial Surgery, Division of Regenerative Orofacial Medicine, University Medical Center Hamburg-Eppendorf, 20246 Hamburg, Germany; 3 Institute for Transfusion Medicine, University Medical Center Hamburg-Eppendorf, 20246 Hamburg, Germany; 4 Department of Periodontics, Preventive and Restorative Dentistry, University Medical Center Hamburg-Eppendorf, 20246 Hamburg, Germany; 5 Department for Vascular Medicine, Translational Wound Research, University Medical Center Hamburg-Eppendorf, 20251 Hamburg, Germany; e.stuermer@uke.de

**Keywords:** oral biofilm, biofilm model, periodontitis bacteria, human plasma, antiseptic solution

## Abstract

**Background/Objectives**: The formation of oral biofilms in periodontal pockets and around dental implants with induction of periodontitis or peri-implantitis is an increasing problem in dental health. The intelligent design of a biofilm makes the bacteria embedded in the biofilm matrix highly tolerant to antiseptic therapy, often resulting in tooth or implant loss. The question therefore arises as to which mouthwashes have eradication potential against oral biofilm. **Methods**: A human oral biofilm model was developed based on donated blood plasma combined with buffy coats, inoculated with oral pathogenic bacterial species found in periodontal disease (*Actinomyces naeslundii*, *Fusobacterium nucleatum*, *Streptococcus mitis*, and *Porphyromonas gingivalis*). Over a span of 7 days, we tested different mouth rinsing and antiseptic solutions (Chlorhexidine, Listerine^®^, NaOCl, Octenisept^®^, and Octenident^®^) covering the matured biofilm with 24 h renewal. Phosphate-buffered saline (PBS) was used as a control. Bacterial growth patterns were detected via quantitative polymerase chain reaction (qPCR) after 2, 4, and 7 days of treatment. **Results**: While all groups showed initial bacterial reduction, the control group demonstrated strong regrowth from day 2 to 4. Listerine showed a near-significant trend toward bacterial suppression. Additionally, strain-specific efficacy was observed, with Octenisept^®^ being most effective against *Streptococcus mitis*, Octenident^®^ and NaOCl showing superior suppression of *Actinomyces naeslundii*, and Listerine^®^ outperforming other solutions in reducing *Fusobacterium nucleatum*. Donor-specific, individual variability further influenced treatment outcomes, with distinct trends in bacterial suppression and regrowth observed across donors. **Conclusions**: These findings underscore the complexity of biofilm-associated infections and highlight the importance of targeted therapeutic approaches for managing bacterial biofilms. In this experiment, the donor-specific outcomes of the antimicrobial effects of the solutions may indicate that genetic predisposition/tolerance to oral infections appears to play a critical role in the control of oral biofilms.

## 1. Introduction

The human oral cavity hosts a complex and diverse microbiota, encompassing both commensal and potentially pathogenic microorganisms. These microbes frequently organize into structured communities embedded within an extracellular matrix of polysaccharides, proteins, and DNA, known as a biofilm. This matrix provides resilience against environmental stressors, antimicrobial agents, and host immune defenses, complicating the management of biofilm-associated infections [1,2]. While biofilms maintain homeostasis under balanced conditions, dysbiosis can lead to diseases such as caries, gingivitis, periodontitis, and peri-implantitis [3,4]. Understanding the dynamics of biofilm formation and its resistance mechanisms is essential for developing effective treatment strategies against oral infections, particularly those associated with periodontal diseases. As biofilm progression occurs, microbial homeostasis can break down, leading to disease [5].

In periodontal disease, excessive plaque accumulation near the gingival margin triggers inflammation, with gingival crevicular fluid introducing host molecules like hemoglobin and transferrin, selecting for proteolytic bacteria. These organisms degrade host regulators of inflammation, leading to tissue damage [5]. Periodontal biofilms exhibit increased biomass, a shift toward Gram-negative anaerobes [6,7], and greater bacterial diversity [5,8,9]. Their interaction with the host immune response promotes inflammation and tissue destruction.

Bacteria within biofilms show reduced susceptibility to antimicrobial agents due to several factors, including limited penetration of agents through the biofilm matrix, altered phenotypes of surface-attached bacteria, and slower bacterial growth rates [10]. Recent studies have highlighted substantial variation in the effectiveness of antimicrobial agents when tested under clinical conditions, such as the extraoral human plasma biofilm model (hpBIOM) [11,12,13]. In these studies, commonly used potent antiseptic solutions and active agents (e.g., sodium hypochlorite (NaOCl), polyhexanide (PHMB), and octenidine-dihydrochloride/phenoxyethanol (OCT/PE)) demonstrated reduced efficacy as biofilm maturation progressed, particularly against *Methicillin-resistant Staphylococcus aureus*, *Pseudomonas aeruginosa* and *Staphylococcus aureus* [11,13], or were rendered ineffective [12].

Current oral biofilm models face limitations in replicating the complexity of the oral environment, including the dynamic interplay between biofilm and host immune defenses [14,15]. Analyses are often carried out on bacterial biofilms that have been colonized on plastic surfaces for only 24 h or that have been cultured in so-called flow chambers without a protein-rich environment or blood cells. These models fail to mimic the physiological microenvironment of human tissues or accurately represent immune competence [11]. To address the challenges, this study builds on the approach of Besser et al. (2019) and (2020), developing a novel oral biofilm model that incorporates human plasma, buffy coat, and four clinically relevant oral bacterial species: *Actinomyces naeslundii*, *Fusobacterium nucleatum*, *Porphyromonas gingivalis*, and *Streptococcus mitis* [11,16]. The use of human plasma can effectively mimic the protein environment found in saliva, as gingival crevicular fluid is a serum exudate rich in host inflammatory mediators, proteins, and ions [17], and approximately 30% of the salivary proteome overlaps with that of plasma [18], making it suitable for studying oral biofilms.

The chosen bacterial strains represent early, intermediate, and late colonizers, reflecting the ecological succession observed in vivo [19]. *Porphyromonas gingivalis*, a keystone pathogen and member of the “red complex”, is strongly associated with periodontitis and peri-implantitis [20]. *Fusobacterium nucleatum*, also linked to these diseases, is known for its strong co-aggregation ability, enhancing the adherence of other bacteria like *Porphyromonas gingivalis* and contributing to the complexity and maturation of the biofilm [21,22]. *Actinomyces naeslundii* supports early biofilm stability by co-aggregating with other species, modulating biofilm acidogenicity, and creating anaerobic conditions that promote microbial growth [23]. *Streptococcus mitis*, a pioneer colonizer, initiates plaque formation by providing binding sites for subsequent microbial attachment [22]. By integrating these species into a plasma-based fibrin matrix, this study aimed to closely simulate the conditions of an inflamed oral environment.

This study evaluated the efficacy in a novel human oral biofilm model (ohpBIOM) of commonly used mouth rinses and antiseptic solutions, including the commercially available products Chlorhexamed FORTE^®^ (containing chlorhexidine digluconate), Listerine^®^ Cool Mint^®^ (essential-oil based), Octenisept^®^ (containing octenidine-hidydrochloride/phenoxyethanol), and Octenident^®^ (containing octenidine), as well as prepared sodium hypochlorite (NaOCl).

## 2. Materials and Methods

### 2.1. Bacterial Strains

*Actinomyces naeslundii* (*A. naeslundii*, DSM no. 17233), *Fusobacterium nucleatum* (*F. nucleatum*, DSM no. 15643), *Porphyromonas gingivalis* (*P. gingivalis*, DSM no. 20709), and *Streptococcus mitis* (*S. mitis*, DSM no. 12643) were obtained from the Leibniz Institute DSMZ–German Collection of Microorganisms and Cell Cultures. All strains were cultured on Schaedler Anaerobe Agar with Sheep Blood, Hemin, Vitamin K1 (Thermo Scientific™, Waltham, MA, USA, catalog no. PB5034A) under anaerobic conditions at 37 °C. The medium used for creating bacterial suspensions contained 15 g/L casein peptone, 5 g/L soy peptone, 5 g/L yeast extract, 5 g/L sodium chloride, 0.5 g/L L-cysteine-HCl, 1% hemin stock solution, 0.1% vitamin K, 10% fetal calf serum (FCS), and 16 g/L agar. For usage, each bacterial suspension was adjusted to a 0.5 McFarland standard (approximately 1.5 × 10^8^ cfu/mL) at 600 nm optical density.

### 2.2. Human Plasma Oral Biofilm Model (ohpBIOM) Preparation

The preparation of this biofilm model was based on a previously described protocol, with minor adjustments regarding model volume and incubation conditions [11,13,16]. Human fresh frozen plasma (FFP) that could not be used clinically due to shelf life and buffy coats was obtained from the Institute for Transfusion Medicine (University Medical Center Hamburg-Eppendorf, Hamburg, Germany). Both components were considered waste products and used in accordance with donor consent for scientific research. Donor selection was random and anonymized, without stratification for age, sex, or health status. FFP (250 mL) was thawed and preserved in a sterile glass bottle. The buffy coat, delivered within a Leucocyte Reduction System (LRS^®^ Cone), was centrifuged for 30 min at 1610× *g* prior to use. Any remaining erythrocytes were removed, and the plasma–leucocyte mix was added to the 250 mL FFP. After mixing at room temperature, the combined bacterial suspension was added, resulting in a final concentration of 2.5 × 10^7^ cells/mL (1.5 × 10^6^ cfu/model). CaCl_2_ (500 mM, 18.26 μL/mL plasma) was then added to the bulk mixture to induce coagulation. The final biofilm mixture was transferred into 12-well culture plates (1.5 mL per well). The well plates were placed in a rotation shaker and incubated for 18 h at 60 rpm under an anaerobic atmosphere at 37 °C for polymerization and biofilm formation. As a result, three biofilm model series were created, each built from a different donor (A, B and C). All models were prepared for quantitative analysis, while one additional model was further analyzed via sequence-induction electron microscopy (see below).

### 2.3. Antimicrobial Treatment of the ohpBIOM and Quantification of Bacterial Load

Five mouth rinsing and antiseptic solutions were used:

- Listerine^®^ Cool Mint^®^ (LIS): Thymol, eucalyptol, methyl salicylate, menthol, water, sorbitol solution, 30% alcohol, poloxamer 407, benzoic acid, sodium saccharin, sodium benzoate, green dye, mint essence (JOHNSON & JOHNSON GmbH D-41470 Neuss, Germany).
- Sodium hypochlorite 1.5% solution (NaOCl): Prepared by mixing 25 g of sodium hypochlorite stock solution (12.5% active chlorine) with 175 g of purified water. Manufactured by the clinic pharmacy, University Hospital Hamburg-Eppendorf, Germany.
- Chlorhexamed FORTE^®^ (CHX): Chlorhexidine bis(D-gluconate) 0.2%, alcohol-free, peppermint aroma, macrogolglycerol hydroxystearate (Ph.Eur.), glycerol, sorbitol-solution (70%) and purified water (GlaxoSmithKline Consumer Healthcare GmbH & Co. KG, 80285 Munich, Germany).
- Octenisept^®^ (OCT): A solution of 0.1% octenidine-dihydrochloride/2% phenoxyethanol (Schülke & Mayr GmbH, Norderstedt, Germany).
- Octenident^®^ mouthwash (OCD): Aqua, PEG-40 hydrogenated castor oil, phenoxyethanol, glycerin, aroma, sodium gluconate, sucralose, citric acid, octenidine HCl, butylhydroxytoluol (Schülke & Mayr GmbH, Norderstedt, Germany).

The agents were used in commercially available concentrations and compared to phosphate-buffered saline (PBS, Gibco^TM^, Thermo Fisher Scientific™, Waltham, MA, USA, Cat. No. 10010023, pH 7.4) treatment, which served as the control. Each well was treated with 100 μL of the mouth rinsing/antiseptic solution starting after 24 h of biofilm formation. The treatment was re-administered every 24 h with the same dosage. After 2, 4, and 7 days of exposure, the samples from each model were processed for quantitative analysis. For Donors A and B, each antiseptic solution was tested in triplicate at days 2, 4, and 7 (technical replicates). Donors A, B, and C represent biological replicates. Untreated controls were included as one sample per solution and time point, resulting in five controls per time point (except at day 0, where only one control served as the baseline). Donor C was tested with single samples per group due to SEM allocation.

For quantitative analysis, bacterial biofilm models were dissolved after antiseptic exposure by adding 1.5 mL (1:1 *v/v*) of a 10% (*w/v*) bromelain solution (Bromelain-POS, URSAPHARM Arzneimittel GmbH, Saarbrücken, Germany) in PBS. The biofilms were detached from the well margins and punctured to improve permeability for enzymatic digestion. Complete dissolution was achieved within 2 h at 37 °C. For DNA quantification, a defined volume of the dissolved samples was harvested for DNA extraction. The volume increase at later timepoints was considered.

### 2.4. Quantitative Polymerase Chain Reaction (qPCR)

Bacterial DNA was isolated according to manufacturer ´s instructions using an innuPREP DNA MiniKit (Analytik Jena AG, Jena/Germany). Pathogen-specific primers were used to identify *Actinomyces naeslundii* (forward: GGTCTCTGGGCCGTTACTGA, reverse: GRCCCCCCACACCTAGTG), *Fusobacterium nucleatum* (forward: AGAGTTTGATCCTGGCTCAG, reverse: GTCATCGTGCACACAGAATTGCTG), *Streptococcus mitis* (forward: GAAGGAGGAGCTTGCTTCTC, reverse: GCGTTGCTCGGTCAGACTTC), and *Porphyromonas gingivalis* (forward: AGGCAGCTTGCCATACTGCG, reverse: ACTGTTAGCAACTACCGATGT). Additionally, universal 16S rRNA primers (eubacteria) were used to detect total bacterial DNA, targeting conserved regions of the 16S rRNA gene (forward: GAGTTTGATCCTGGCTCAG, reverse: GWATTACCGCGGCKGCTG).

The qPCR was performed in a CFX96™ Real-Time System (Bio-Rad, Hercules, CA, USA) with the Luna^®^ Universal qPCR Master Mix (New England Biolabs) for fluorescence detection. Each 20 μL reaction contained 2 μL template DNA, 1 μL of primer mix, and 7 μL of nuclease-free water to adjust the final volume. The thermal cycling protocol included using 37 °C for 25 min, 95 °C for 15 min, 35 cycles of 95 °C for 15 s, 60 °C for 20 s, 65 °C for 20 s, 77 °C for 1 s, followed by melt curve analysis from 60 °C to 95 °C with 0.5 °C increments.

Positive controls included DNA from the respective bacterial strains (*Actinomyces naeslundii* DSM17233, *Fusobacterium nucleatum* DSM15643, *Porphyromonas gingivalis* DSM20709, and *Streptococcus mitis* DSM12643). No-template controls (NTC) were included in each qPCR run to monitor for contamination. Data were analyzed using Bio-Rad CFX Maestro (v2.3), and amplification curves and melting curves were assessed to confirm specificity.

### 2.5. Scanning Electron Microscopy (SEM)

Following the protocol by Besser et al., 2020 [11], the ohpBIOM samples were fixed in 0.1 M cacodylate buffer containing 2.5% glutaraldehyde, 2% polyvinylpyrrolidone, and 75 mM NaNO_2_ for 1 h at 4 °C. After fixation, the samples were rinsed in 0.1 M cacodylate buffer without glutaraldehyde. The biofilm models were then cut into small pieces by freezing in liquid nitrogen to expose both surface and cross-sectional areas. Glycocalyx staining was performed by immersing the samples in a solution of 2% arginine-HCl, glycine, sucrose, and sodium glutamate for 18 h at room temperature (RT). The biofilms were washed with distilled water and subsequently immersed in a mixture of 2% tannic acid and 2% guanidine-HCl for 5.5 h at RT. After rinsing with distilled water, the samples were incubated in a 1% OsO_4_ solution for 30 min at RT. Following three additional rinses with distilled water, the specimens were dehydrated through a graded ethanol series and dried using liquid CO_2_ within a critical-point dryer. The dried specimens were mounted onto SEM stubs using carbon adhesive stickers and secured with silver glue for improved conductivity. The samples were then sputter-coated with gold-palladium and examined using a Zeiss Sigma SEM (Zeiss, Oberkochen, Germany) at an acceleration voltage of 2 kV. Due to morphological variation and technical imaging constraints, such as differing working distances and surface conductivity, SEM images were captured at varying settings. This can result in differences in resolution even at similar magnifications. Since the SEM analysis was qualitative, we selected representative images that offered the best visual contrast and surface detail. All images include visible scale bars to support interpretation.

### 2.6. Statistical Analysis

As part of the analysis, an initial descriptive exploration of the data was conducted to summarize key characteristics and distributions. Inferential analyses were subsequently performed using a generalized linear model (GLM). The dependent variable was the Log10-transformed bacterial count, chosen to normalize the exponential distribution and stabilize variance. Fixed effects in the model included the type of solution and the specific bacterial strain under investigation. The total bacterial count was additionally modeled as an alternative strain to provide a comprehensive comparison. Donor variability was incorporated as a random effect to account for inter-individual differences. A compound symmetry covariance structure was applied to model the correlation between repeated measurements effectively.

Data processing, data analysis, and the creation of figures were performed using SAS Software version 9.4 (SAS Institute Inc., Cary, NC, USA). A significance threshold of 5% was set for Type I error, ensuring that results with *p*-values below 0.05 were considered statistically significant. Given the exploratory nature of this study, no adjustments were made for multiple testing across the analyses.

Post hoc test results were selectively presented, even in instances where the omnibus test failed to reach statistical significance. In these cases, *p*-values were marked as p*, explicitly indicating their non-confirmatory nature. Such results should be interpreted with caution and are intended to provide a basis for hypothesis generation and subsequent research rather than definitive conclusions.

Microscopic analysis was descriptive and qualitative without statistical considerations.

## 3. Results

### 3.1. Overall Bacterial Growth Trends (Donors A–C)

Our study evaluated the effects of various mouth rinsing and antiseptic solutions, including a control group (PBS), on bacterial load in a biofilm model derived from human plasma and buffy coat from three donors (A, B, and C), representing varying immune competencies. The biofilm model incorporated four typical oral bacterial species and was designed to mimic complex microbial communities. Measurements of bacterial load were conducted at days 2, 4, and 7 using qPCR analysis.

In this analysis, the global (omnibus) test revealed statistically significant effects for day (*p* < 0.0001) and donor (*p* = 0.0077) on bacterial load. However, no eradication effect was found for mouth rinsing/antiseptic solutions (*p* = 0.3923), nor for the interaction between the solution and day (*p* = 0.1468)—in contrast to their proven efficacy against the planktonic bacteria often used in tests of the minimum inhibitory concentration (MIC) [24,25,26]. These findings suggest that bacterial survival was primarily influenced by time and donor variability rather than the solution itself. Based on these results, post hoc pairwise comparisons are presented in an exploratory manner, and *p*-values from these comparisons are reported as p*. These exploratory p*-values are intended to highlight potential trends in bacterial suppression associated with specific treatments.

**Figure 1:**
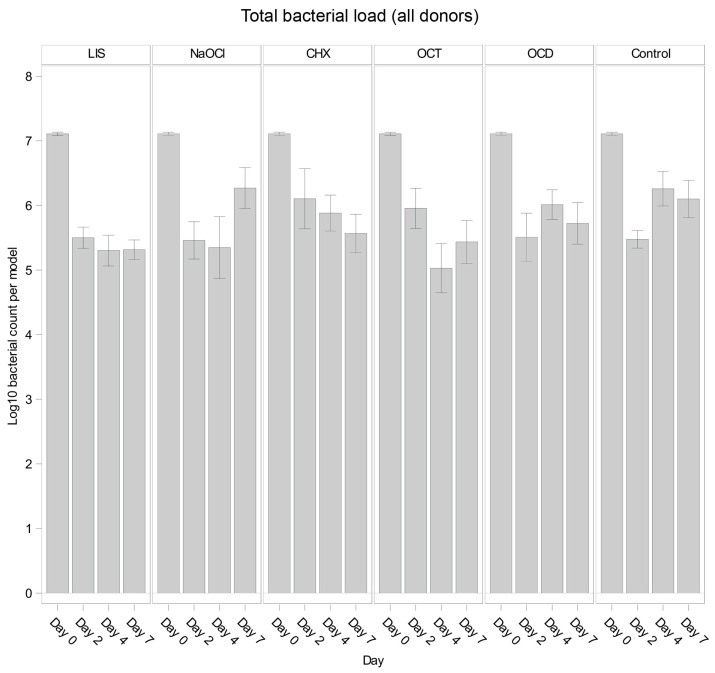
Comparison of bacterial reduction in human oral multi-species biofilm models (ohpBIOM) across different donors after application of five different mouth rinsing and antiseptic solutions (Chlorhexidine (CHX), Listerine (LIS), NaOCl, Octenident (OCD), Octenisept (OCT)) in a prolonged exposure of up to 7 days. Graphs depict bacterial load (in log10 bacterial count per model) compared to an untreated control (Control). Values are expressed as means +/− SD. No significant differences were found.

Figure 1 illustrates the overall trends in total bacterial load for each solution and control. Across all oral biofilms, bacterial load decreased significantly from day 0 to day 2 (*p* < 0.001) for all groups, including the control, likely reflecting early biofilm dynamics. However, subsequent trends varied. The control group showed bacterial regrowth from day 2 to day 4 (*p* = 0.0189 *), followed by stabilization from day 4 to day 7 (*p* = 0.6324 *), suggesting that bacterial growth plateaued in the absence of treatment.

Among the tested solutions, LIS demonstrated consistent bacterial reductions through day 4 (*p* = 0.0040 * from day 0 to day 2), remaining largely unchanged until day 7 (*p* = 0.9733 *). Similarly, OCT reduced bacterial load significantly until day 4 (*p* = 0.0268 *), showing a minor rebound by day 7. In contrast, NaOCl exhibited minor reductions by day 4 (*p* = 0.7885 *) but showed noticeable bacterial regrowth by day 7 (*p* = 0.0277 *), surpassing the control levels. OCD and CHX showed overall less reductions compared to LIS, OCT, and NaOCl, while OCD followed a trend comparable to the control and CHX displayed a slight decrease of bacterial load at each timepoint but without statistical efficacy.

### 3.2. Overall Efficacy of Tested Solutions Compared to Control

Across all time points, the least squares mean for bacterial load showed variability among the tested solutions and the control. While LIS (Log10 5.837) exhibited the strongest trend toward reducing bacterial load relative to the control, followed by OCT (Log10 5.912) across the experiment, no solution showed a statistically significant overall effect compared to the control when evaluated over all time points combined (*p* > 0.05 for all comparisons). Notably, LIS demonstrated the largest numerical difference from the control with an estimate close to the threshold for significance (*p* = 0.0556 *). Other solutions, such as NaOCl, CHX, and OCD, were not distinguishable in anti-bacterial efficacy from the control when assessed across the full experimental timeline.

In intergroup comparisons, none of the solutions showed a significant difference compared to the untreated control on day 2, with *p*-values of 0.9439 *, 0.2125 *, 0.1008 *, 0.9683 *, and 0.9531 * for NaOCl, OCT, CHX, LIS, and OCD, respectively. On day 4, NaOCl (*p* = 0.0151 *), OCT (*p* = 0.0012 *), and LIS (*p* = 0.0108 *) showed higher trends of bacterial reduction in the oral biofilms while CHX (*p* = 0.3026 *) and OCD (*p* = 0.4924 *) showed no notable differences. By day 7, only LIS demonstrated a trend toward bacterial growth reduction (*p* = 0.0354 *), while NaOCl (*p* = 0.6745 *), OCT (*p* = 0.0732 *), CHX (*p* = 0.1487 *), and OCD (*p* = 0.3015 *) had no remarkable effect.

These results indicate variability in the efficacy of mouth rinsing and antiseptic solutions and highlight that while NaOCl, OCT, and LIS showed trends of reducing bacterial loads in the oral biofilm until day 4, only LIS maintained a control of bacterial regrowth up to day 7. The control group demonstrated an early bacterial reduction within the first 48 h followed by regrowth, consistent with sustained biofilm formation.

### 3.3. Donor-Specific Differences

**Figure 2:**
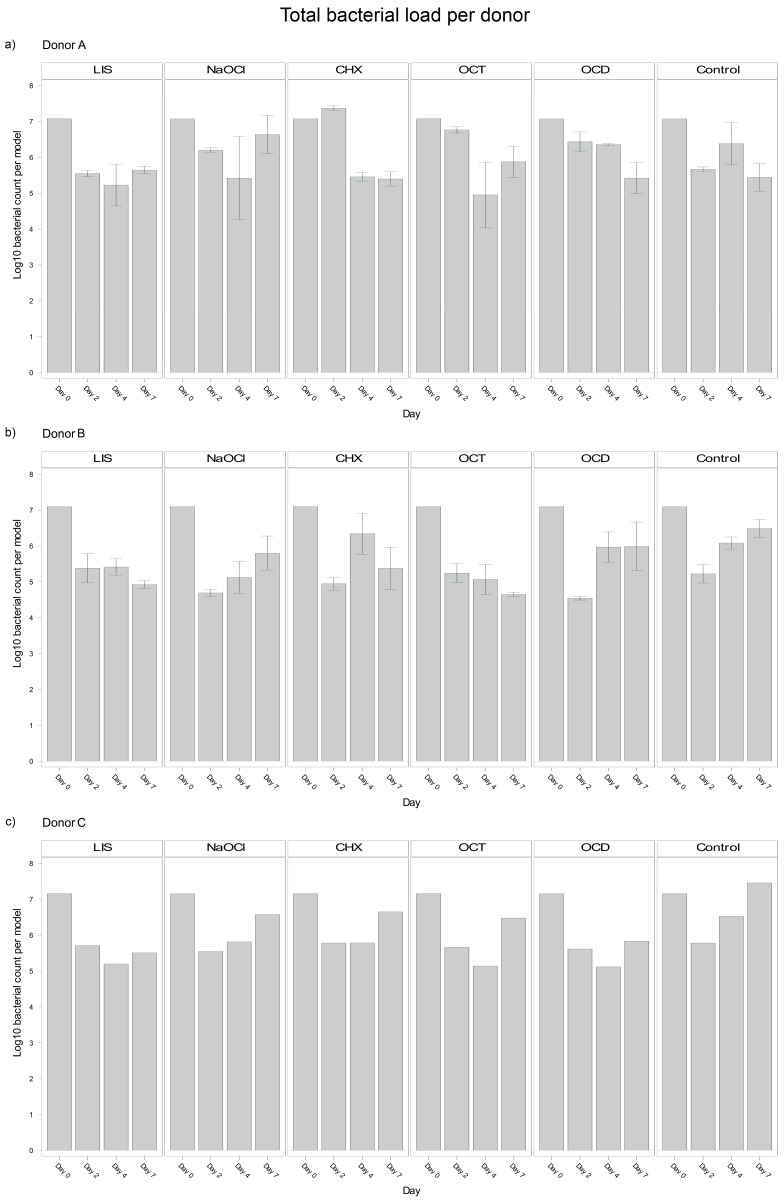
Donor-specific variability of bacterial reduction in human oral multi-species biofilm models (ohpBIOM) after application of five different mouth rinsing and antiseptic solutions (Chlorhexidine (CHX), Listerine (LIS), NaOCl, Octenident (OCD), Octenisept (OCT)) in a prolonged exposure of up to 7 days. Graphs depict bacterial load (in log10 bacterial count per model) compared to an untreated control (Control). (**a**) Bacterial count for Donor A; (**b**) Bacterial count for Donor B; (**c**) Bacterial count for Donor C. Values are expressed as means +/− SD.

Analysis of blood donor-specific bacterial load in the oral biofilm models revealed significant differences between Donors A and B (*p* = 0.0040), and Donors B and C (*p* = 0.0295) with Donor B exhibiting a smaller bacterial load (Log10 5,8). However, no significant difference was observed between Donors A and C (*p* = 0.9261). These differences suggest that individual donor variability influenced bacterial load outcomes in the biofilms, potentially reflecting differences in the immune competence or other donor-specific factors incorporated in the plasma and/or buffy coat. Despite the overall trends discussed above, notable donor-specific differences could be observed (Figure 2).

LIS maintained a reduction of bacterial load across all donors but showed a stronger trend in Donor B, with bacterial load continuing to decline between days 4 and 7 (Log10 5.413 → 4.924). Donors A and C exhibited slight bacterial regrowth during the same period (Log10 5.226 → 5.644 and Log10 5.198 → 5.508, respectively).

NaOCl resulted in bacterial suppression by day 4 across donors but exhibited significant regrowth by day 7. Donor A displayed the strongest rebound (Log10 5.419 → 6.635), followed by Donor C (Log10 5.812 → 6.575).

OCT was effective at reducing bacterial load across all donors, with notable reductions by day 4. However, within Donor A and C, bacteria regrew from day 4 to day 7 (Log10 4.953 → 5.876 and Log10 5.139 → 6.477 respectively). Donor B, in contrast, demonstrated continued reductions, with bacterial load decreasing continuously from day 0 to day 7 (Log10 7.096 → 4.649).

CHX exhibited the most donor-specific variability. In Donor A, CHX showed an initial increase by day 2 (Log10 7.074 → 7.368), followed by a strong decline by day 4 and stabilization by day 7 (Log10 5.396). Donor B exhibited a sharp reduction by day 2 (Log10 7.096 → 4.949), followed by regrowth through day 4 and slight stabilization by day 7 (Log10 5.380). In Donor C, consistent bacterial regrowth started after day 4, surpassing the initial reductions by day 7 (Log10 5.784 → 6.649).

OCD showed steady reductions for Donor A throughout the study period (Log10 7.074 → 5.421), while Donor B exhibited regrowth between day 2 and 7 (Log10 4.545 → 5.989). Donor C showed almost no changes from day 4 to day 7 (Log10 5.116 → 5.833).

These observations show an interplay between bacteria, donor immunity and the tested mouth rinsing and antiseptic solutions, where biofilm models from different donors exhibited different bacterial suppression patterns despite the same treatment. Nevertheless, the most effective solutions tested, LIS and OCT, maintained a consistent performance trend across all donors, whereas CHX showed a large variability.

### 3.4. Strain-Specific Antimicrobial Efficacy

To assess differences of antimicrobial efficacy on the selected strains, qPCR with the respective primers was performed for detection of *A. naeslundii*, *F. nucleatum*, *P. gingivalis* and *S. mitis*. The following figures show the strain-specific outcomes for Donor A and B compared to the mean of all donors. Statistical analysis revealed significant effects of mouth rinsing and antiseptic solutions regarding the bacterial load of *A. naeslundii* (*p* < 0.0001), *F. nucleatum* (*p* = 0.0008), and *S. mitis* (*p* = 0.0342) but not for *P. gingivalis* (*p* = 0.2525). Notably the efficacy of mouth rinsing and antiseptic solutions differed among the selected strains. Donor variability and the duration of biofilm formation also had significant impacts on treatment outcomes (*p* < 0.0001).

#### 3.4.1. *Actinomyces naeslundii*

**Figure 3:**
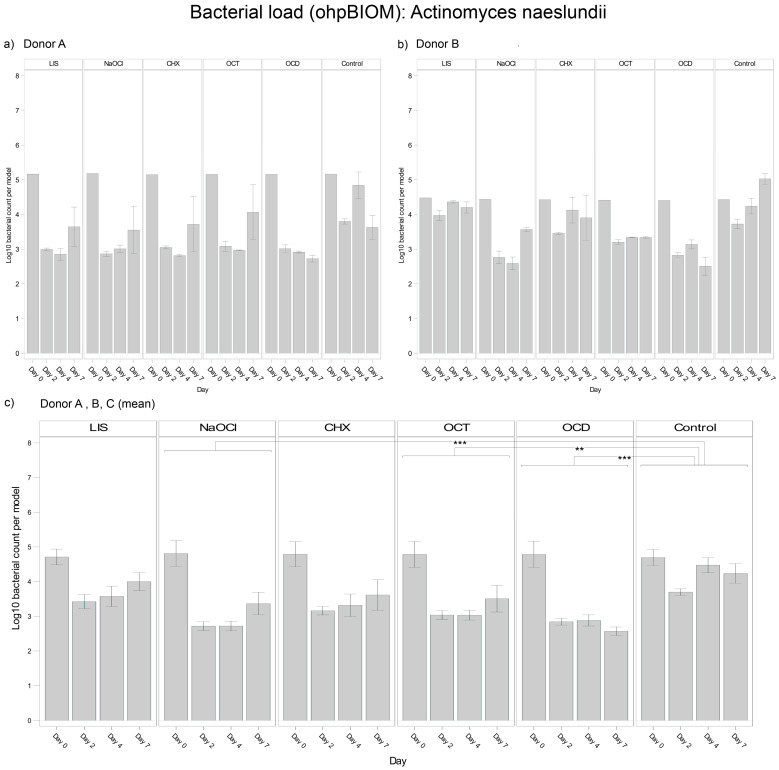
Strain- and donor-specific variability—(**a**) Donor A; (**b**) Donor B; (**c**) Mean of all donors—of *A. naeslundii* reduction in human oral multi-species biofilm models (ohpBIOM) after application of five different mouth rinsing and antiseptic solutions (Chlorhexidine (CHX), Listerine (LIS), NaOCl, Octenident (OCD), Octenisept (OCT)) in a prolonged exposure of up to 7 days. Graphs depict bacterial load (in log10 bacterial count per model) compared to an untreated control (Control). Values are expressed as means +/− SD. Significant differences between the solutions and control group are marked with ** *p* < 0.01 and *** *p* < 0.001. OCD and NaOCl demonstrated the strongest effects on the reduction of *A. naeslundii*, while the effect showed variability among individual donors.

For reduction of *A. naeslundii*, OCD (Log10 3.111) and NaOCl (Log10 3.245) were the most effective solutions, followed by OCT (Log10 3.431) and CHX (Log10 3.562). These solutions demonstrated statistically significant reductions in bacterial load compared to the control (Log10 4.148), with *p*-values of <0.0001, <0.0001, 0.0002, and 0.0018, respectively. In contrast, LIS showed no statistically relevant reduction of the bacterial load but exhibited different trends of efficacy among donors (Figure 3). In general, bacterial growth of *A. naeslundii* varied slightly among different donors.

#### 3.4.2. *Fusobacterium nucleatum*

**Figure 4:**
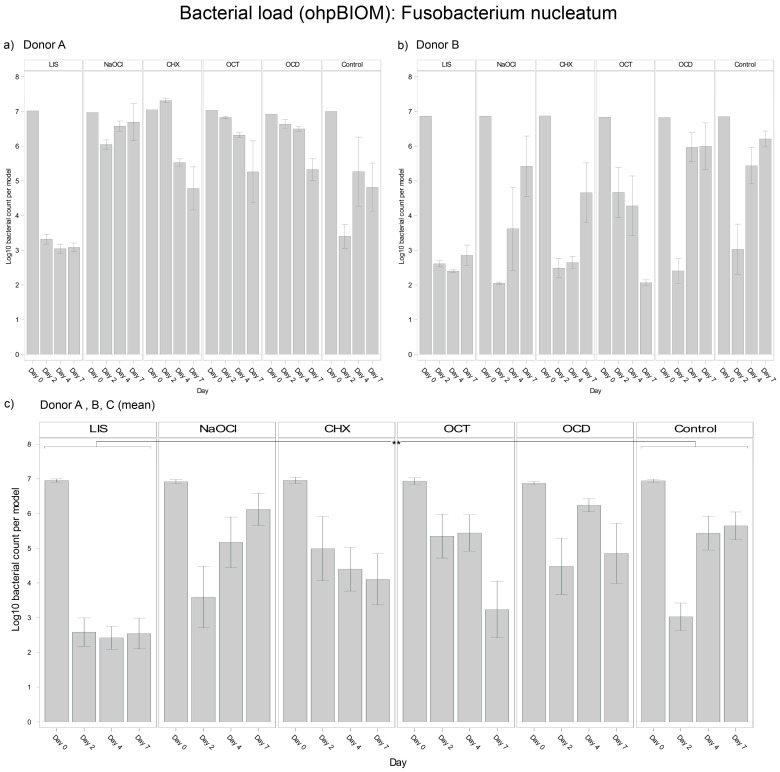
Strain- and donor-specific variability—(**a**) Donor A; (**b**) Donor B; (**c**) Mean of all donors—of *F. nucleatum* reduction in human oral multi-species biofilm models (ohpBIOM) after the application of five different mouth rinsing and antiseptic solutions (Chlorhexidine (CHX), Listerine (LIS), NaOCl, Octenident (OCD), Octenisept (OCT)) in a prolonged exposure of up to 7 days. Graphs depict bacterial load (in log10 bacterial count per model) compared to an untreated control (Control). Values are expressed as means +/− SD. Significant differences between the solutions and control group are marked with ** *p* < 0.01. LIS demonstrated the strongest effect on the reduction of *F. nucleatum*.

For reduction of *F. nucleatum*, LIS (Log10 3.467) was the most effective solution, achieving the lowest bacterial load in the oral biofilm compared to all other treatments (*p* = 0.0003) (Figure 4). NaOCl (Log10 5.201) and OCD (Log10 5.360) were the least effective, with no effects compared to the control (Log10 5.061). OCT (Log10 4.989) and CHX (Log10 4.863) showed slightly lower bacterial reductions overall but achieved significant reductions compared to the control on day 7 (*p* = 0.0010 and *p* = 0.0364, respectively). Differences among donors were pronounced in the CHX and control groups, with *F. nucleatum* decreasing in oral biofilms of Donor A but regrowing in Donors B and C. The consistency of LIS efficacy across donors underscores its superior efficacy against *F. nucleatum*.

#### 3.4.3. *Porphyromonas gingivalis*

**Figure 5:**
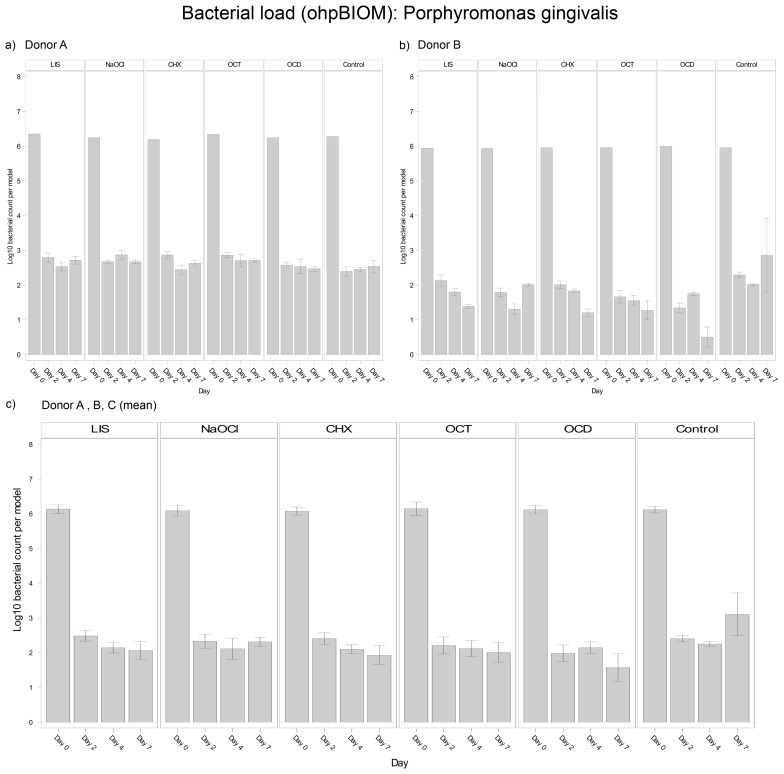
Strain- and donor-specific variability—(**a**) Donor A; (**b**) Donor B; (**c**) Mean of all donors—of *P. gingivalis* reduction in human oral multi-species biofilm models (ohpBIOM) after application of five different mouth rinsing and antiseptic solutions (Chlorhexidine (CHX), Listerine (LIS), NaOCl, Octenident (OCD), Octenisept (OCT)) in a prolonged exposure of up to 7 days. Graphs depict bacterial load (in log10 bacterial count per model) compared to an untreated control (Control). Values are expressed as means +/− SD. OCD demonstrated the strongest effect on the reduction of *P. gingivalis* without statistical significance.

For reduction of *P. gingivalis*, OCD (Log10 3.029) demonstrated the greatest bacterial suppression; however, it was not statistically relevant overall in terms of reducing the bacterial load compared to the control (Log10 3.530; p* = 0.0166) (Figure 5). The other solutions, LIS (Log10 3.255), CHX (Log10 3.203), OCT (Log10 3.195) and NaOCl (Log10 3.287), showed comparable reductions among each other without significant differences. Trends were relatively consistent across donors, although bacterial regrowth from day 4 to day 7 was more pronounced in Donor C.

#### 3.4.4. *Streptococcus mitis*

**Figure 6:**
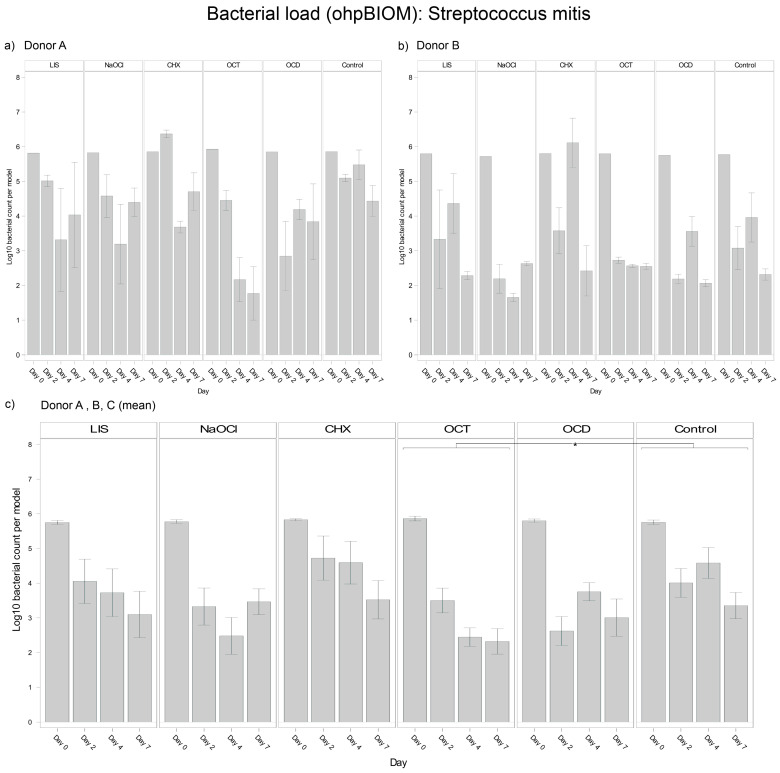
Strain- and donor-specific variability—(**a**) Donor A; (**b**) Donor B; (**c**) Mean of all donors—of *S. mitis* reduction in human oral multi-species biofilm models (ohpBIOM) after application of five different mouth rinsing and antiseptic solutions (Chlorhexidine (CHX), Listerine (LIS), NaOCl, Octenident (OCD), Octenisept (OCT)) in a prolonged exposure of up to 7 days. Graphs depict bacterial load (in log10 bacterial count per model) compared to an untreated control (Control). Values are expressed as means +/− SD. Significant differences between the solutions and control group are marked with * *p* < 0.05. OCT demonstrated the strongest effect on the reduction of *S. mitis*.

For reduction of *S. mitis*, OCT (Log10 3.438) achieved the greatest reduction of the bacterial load (*p* = 0.0155 vs. control, Log10 4.349) (Figure 6). NaOCl (Log10 3.666) and OCD (Log10 3.701) demonstrated comparable efficacy. CHX exhibited the least efficacy, resulting in a higher overall *S. mitis* load in the oral biofilms and being statistically less effective than OCT, OCD, and NaOCl. LIS (Log10 4.210) showed less *S. mitis* reduction, with bacterial loads slightly lower than the control but without achieving statistical significance. While control group trends were similar across donors, the extent of bacterial reduction varied slightly, reflecting donor-specific dynamics.

In summary, the antibacterial efficacy of the tested mouth rinsing and antiseptic solutions varied significantly across bacterial strains: OCD consistently emerged as the most effective solution for *A. naeslundii*, while LIS showed superior performance against *F. nucleatum*, outperforming all other solutions with consistent trends across donors. OCT was most effective against *S. mitis* with significant reductions also observed for *A. naeslundii*. CHX demonstrated moderate to limited antibacterial effects, with a performance dependent on the donor and strain. These findings highlight the importance of considering both strain-specific antimicrobial efficacy and donor variability in interpreting bacterial suppression and biofilm resilience. However, comparisons in total bacterial reduction remained non-significant, resulting in limited antimicrobial efficacy against oral biofilms.

### 3.5. Scanning Electron Microscopy Analysis

Scanning electron microscopy (SEM; Zeiss Sigma, Germany) was used to evaluate the structural and compositional integrity of the biofilm model under different conditions. Following glycocalyx staining and sample preparation as described above, the biofilm morphology was assessed at magnifications of ×500 to ×15,000.

**Figure 7:**
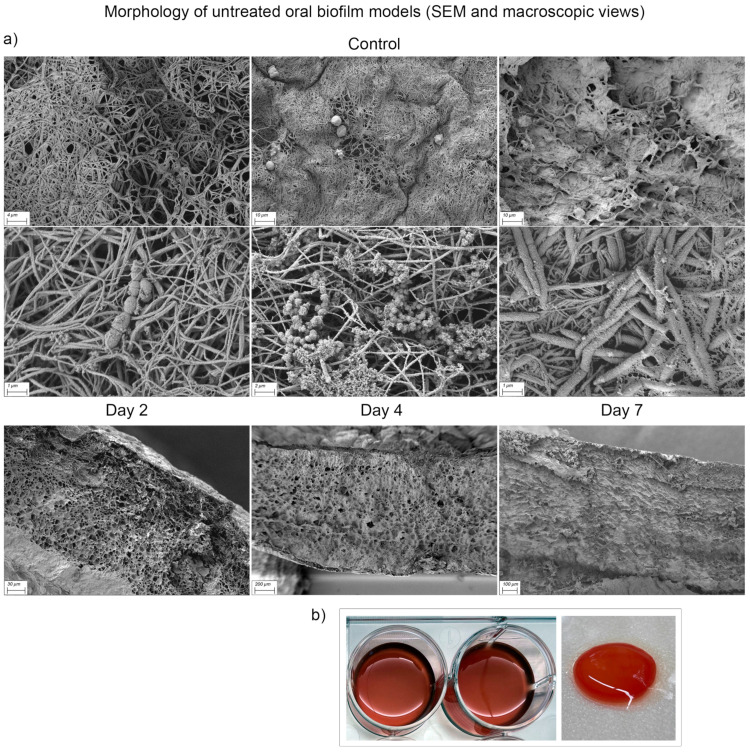
Scanning electron microscopy (SEM) and macroscopic visualization of multi-species oral biofilm model surface (ohpBIOM) without treatment (Control) on days 2–7. (**a**) Different bacteria and biofilm formation can be observed in the SEM images. Loose, fibrin-dominated surface structure on day 2 tends to consolidate into a dense, kit-like surface by day 7. Extracellular polymeric substance surrounding the bacteria increases with each timepoint. The lower series of images shows the cross-section of the Control biofilm models with increasing density. (**b**) Macroscopic appearance of ohpBIOM. During fibrin polymerization, the model consolidates and offers a three-dimensional matrix for biofilm formation.

The untreated mature oral biofilm model (day 2) exhibited a dense, interconnected structure of fibrin mesh with a fine developing glycocalyx encapsulating the bacteria (Figure 7). Micro colonies on the surface were clearly visible, although sporadically distributed. Residual human blood cells embedded in the fibrin matrix were observed at all time points. As the biofilm consolidated, bacteria became increasingly surrounded by extracellular polymeric substances (EPS), forming a continuous and stable biofilm matrix. Cross-sectional imaging revealed the multilayered architecture of the biofilm model: During early biofilm maturation, a net-like structure with numerous pores and interstices was prominent. Over time, progressively denser structures with kit-like closure of the interstices became apparent.

**Figure 8:**
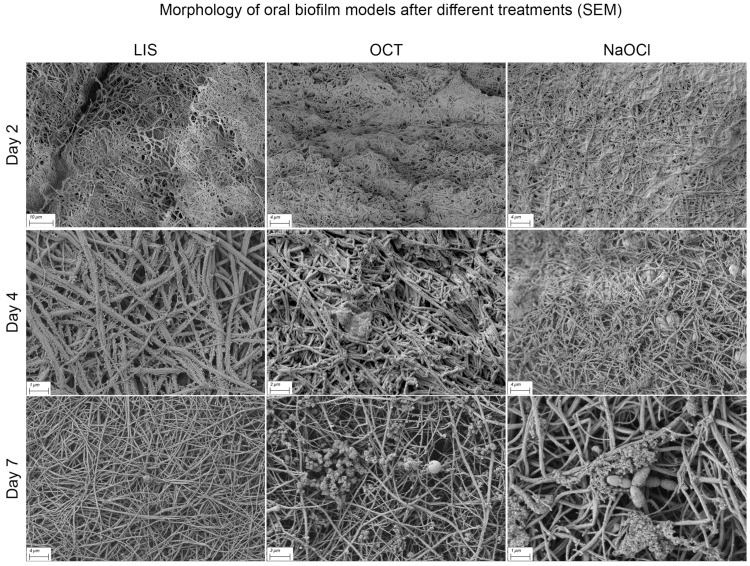
Scanning electron microscopy (SEM) visualization of multi-species oral biofilm model surface alteration in the ohpBIOM under different treatment conditions (LIS, OCT and NaOCl) on days 2–7. The depicted solutions appear to interact with the extracellular polymeric substance and surface proteins, resulting in a more exposed fibrin structure on the surface compared to the control on day 7 (see Figure 7).

Differences in biofilm structure according to treatment modalities were not distinct. However, with increasing treatment duration, the fibrin surface became more visible, particularly with LIS, NaOCl and OCT, indicating a possible interaction with EPS and surface proteins (Figure 8). Occasionally, honeycomb-like pores at the cross-sectional margin were observed, but their origin could not be definitively determined. The biofilm matrix surface showed varying levels of deposited material, potentially remnants or components of human plasma and blood cells.

Among the tested solutions, none demonstrated a potent ability to completely penetrate and disrupt the biofilm matrix. A trend toward reduced EPS on the biofilm surface was observed for certain treatments. Conversely, CHX appeared to have a minimal effect, with the biofilm structure remaining largely intact even after prolonged exposure.

## 4. Discussion

To our knowledge, this is the first systematic and comparative study to evaluate the efficacy of commonly used mouth rinsing and antiseptic solutions in a 3D human plasma-based oral biofilm model designed to mimic the complexity of periodontal biofilms. The model incorporated key features of oral biofilms, including bacterial diversity, extracellular polymeric substance (EPS) formation, and the host-derived components from human plasma as well as the individual immune competence (buffy coat). Existing basic research on oral biofilm models, which aim to understand bacterial interactions and to develop and test therapeutic strategies against periodontitis or peri-implantitis, focus on the reconstruction of the hydrodynamic and physicochemical microenvironment of the oral cavity, monitoring flow, shear forces and pH or using artificial/human saliva for biofilm growth [27,28,29]. They often neglect human cellular and acellular components, such as immune competence, that constantly challenge biofilm formation. Therefore, the commonly used term ‘oral biofilm model’ is not uniformly defined. Biofilm models resulting from planktonic bacteria in an artificial medium with/without a flow chamber are used as in vitro biofilm models [30,31]. Furthermore, models typically use attachment and growth of bacteria on adherent materials such as plastic wells [32], glass slides, hydroxyapatite discs [31] or metallic surfaces [33], while different systems, such as chemostats, drip flow reactors or flow cell systems facilitate the growth of the biofilm [14,34,35]. Recently, an oral in vitro biofilm model cultured on 3D substrate has been proposed, supporting multidirectional biofilm growth resulting in a higher biomass [36]. Unfortunately, all these models lack important factors for the clinical reality of oral inflammation and infection: the human physiological oral environment, the local immune competence and bacterial diversity.

The human plasma-based biofilm model employed in this study offers several advantages, including the incorporation of host-derived components that mimic the physiological conditions of an inflamed oral environment. The established microenvironment can simulate inflammatory conditions characterized by increased gingival crevicular fluid flow or mimic post-surgical scenarios, where pathogens are exposed to a wound site after active bleeding. Although the presence of plasma proteins, such as fibrinogen, is known for its inhibitory effect on co-aggregation of certain oral bacteria [37,38], it supports biofilm structure by providing a three- dimensional matrix, creating niches for biofilm development. Moreover, the assumed initial immune activity in the early phase demonstrates the model’s ability to replicate host–pathogen interactions to a certain degree, providing valuable insights into antimicrobial efficacy under biologically realistic conditions. While the immune cell activity in later anaerobic stages is limited, it also reflects the challenges faced in chronic biofilm-associated infections, where the immune system may be compromised. This emphasizes the clinical relevance of evaluating antiseptic agents under such conditions.

The initial reduction in bacterial load observed across all groups, including the control, likely reflects early immune activity associated with the buffy coat-derived leukocytes. These immune cells, particularly neutrophils, are known for their antimicrobial capabilities through phagocytosis, oxidative bursts, and antimicrobial peptide release [39]. These findings align with previous research emphasizing the role of leukocytes in early biofilm disruption [11]. However, as anaerobic conditions dominate in the later experimental stages, immune cell viability or function likely diminishes, leading to bacterial regrowth, particularly in the control group.

According to the systematic review and meta-analysis by Ramanauskaite et al. (2021), chlorhexidine (CHX) and sodium hypochlorite (NaOCl) are among the commonly investigated antiseptics used adjunctively in the non-surgical treatment of peri-implant mucositis and peri-implantitis [40]. However, their clinical benefits remain inconsistent across studies. In peri-implant mucositis, CHX was applied either locally as a gel [41] or through a full-mouth disinfection protocol [42,43]. Most studies found no significant improvements in bleeding on probing (BOP) or probing depth (PD) when compared to mechanical debridement alone. Only one trial reported a greater reduction in PD when using CHX gel [41]. NaOCl, which was evaluated as a topically applied gel in one study [44], similarly showed no added clinical benefit. Notably, while NaOCl is widely used in root canal treatment [45,46], its use in peri-implant therapy is limited. In peri-implantitis therapy, CHX was often included in control protocols but showed no additional advantage over mechanical or other adjunctive treatments [40]. An in vitro study reported MIC values of chlorhexidine against *P. gingivalis* ranging from 60 to 120 µg/mL under anaerobic conditions [47]. Another in vitro biofilm study using a 0.12% chlorhexidine solution (1200 µg/mL) showed complete elimination of *P. gingivalis* and significant reduction of *F. nucleatum* levels in a multispecies setting [48]. While our study used a 0.2% CHX formulation, it showed no significant antimicrobial effect on these strains, aligning with clinical studies that report limited benefit of adjunctive CHX, particularly in peri-implant therapy.

Overall, while CHX and NaOCl have been investigated in peri-implant therapy, neither demonstrates consistent clinical benefit in adjunctive use, which is consistent with the results obtained here. As such, their routine use—particularly in non-surgical settings—is not recommended in current German guidelines [49]. This assessment is supported by the 2023 EFP S3-level clinical practice guideline [50], which explicitly advises against the use of chlorhexidine (or photodynamic therapy) for implant surface decontamination during surgical peri-implantitis treatment, citing very low certainty of evidence. Similarly, in non-surgical treatment of peri-implant mucositis, adjunctive CHX or NaOCl seemed to have no clinical advantage over mechanical debridement alone [50].

For the treatment of periodontitis, CHX remains a widely used adjunctive antiseptic agent. The meta-analysis by da Costa et al. (2017) reported that adjunctive CHX mouthwash following scaling and root planing (SRP) resulted in statistically significant but clinically modest reductions in PD, with minimal gains in clinical attachment level [51]. The EFP S3 Clinical Practice Guideline by Sanz et al. (2020) aligns with these findings, issuing an open recommendation (Grade 0) for the use of CHX, reflecting weak support for routine adjunctive application. The guideline emphasizes mechanical biofilm removal as the cornerstone of effective periodontal therapy [52].

Taken together, the evidence for CHX and NaOCl in both peri-implant and periodontal therapy suggests that mechanical instrumentation should remain the primary therapeutic approach to disrupting biofilms, with antiseptic adjuncts used selectively rather than routinely.

Regarding Listerine and octenidine-based solutions (OCT and OCD), several clinical trials have demonstrated their efficacy in reducing dental plaque and improving gingival health [53,54]. A common clinical approach to evaluate antiseptic efficacy involves collecting saliva or rinse samples before and after mouth rinsing, followed by incubation and comparison of colony-forming units (CFU) [55,56]. In contrast, in vitro studies often assess antimicrobial effects against planktonic bacteria [57,58,59], which may not accurately represent biofilm-associated resistance. While an early in vitro study by Kato et al. (1990) reported that Listerine inhibited oral pathogens such as *F. nucleatum*, *P. gingivalis*, *A. naeslundii*, and *S. mitis* at dilutions of 6.25 to 12.5% in suspension cultures [60], a more recent study by Siddiqui et al. (2025) evaluated Listerine^®^ Cool Mint^®^ in a multispecies biofilm model and found no significant reduction in *F. nucleatum* or *P. gingivalis*, highlighting the limited efficacy of Listerine against these strains under biofilm conditions [48]. A recent preprint study reported that an octenidine-based mouthwash exhibited strong antimicrobial activity against *P. gingivalis* and *F. nucleatum*, with MICs of 0.25 µg/mL, while *A. naeslundii* showed reduced sensitivity (MIC 2.0 µg/mL) under planktonic conditions [61]. In contrast, our study using OCD (0.1%) in a mature biofilm model showed limited efficacy against *F. nucleatum* but a more pronounced effect on *A. naeslundii*.

Despite evaluating a range of antiseptic agents, none of the tested solutions demonstrated statistically significant reductions in total bacterial load compared to the untreated control across the experimental timeline. Listerine (LIS) exhibited the strongest trend toward bacterial suppression, with a near-significant outcome (*p* = 0.0556). This effect might be attributed to its active ingredients, including thymol, menthol, and eucalyptol, which are known to disrupt bacterial membranes and interfere with EPS formation [62]. Sodium hypochlorite (NaOCl) and Octenisept (OCT) exhibited strong reductions by day 4 but showed varying rebound effects by day 7, depending on the donor. SEM imaging suggested that while these solutions interacted with the biofilm matrix, complete structural disruption was not achieved. Chlorhexidine (CHX) showed limited efficacy, with variable outcomes across donors. This variability may be due to the protective properties of mature biofilms, which shield bacteria from antimicrobial agents [63].

While total bacterial load outcomes were inconclusive, significant differences were observed when targeting individual bacterial strains. OCD and NaOCl were highly effective against *Actinomyces naeslundii*, while LIS outperformed other treatments for *Fusobacterium nucleatum*. OCT demonstrated the greatest suppression of *Streptococcus mitis*, and OCD was most effective against *Porphyromonas gingivalis*. These strain-specific differences underline the potential use of targeted therapeutic strategies that consider the unique susceptibilities of individual pathogens within biofilm communities.

Donor-specific variability played a significant role in treatment outcomes. These differences likely reflect variations in immune competence and plasma composition among donors, emphasizing the potential value of personalized treatment approaches in biofilm-associated infections. Despite this variability, solutions like LIS and OCT maintained relatively consistent trends across all donors, while CHX displayed greater variability in efficacy. These findings emphasize the importance of further research into host-specific factors influencing biofilm resilience and treatment outcomes to develop individual treatment strategies.

The observed species-specific differences in bacterial reduction further highlight the complex interplay between microbial composition in biofilms and treatment efficacy. Supporting these findings, Mikolai et al. (2020) demonstrated that bacterial species within biofilms exhibit distinct interactions with immune cells, such as neutrophils [64]. For example, *Porphyromonas gingivalis* employs evasion strategies to suppress immune responses, whereas *Fusobacterium nucleatum* contributes to biofilm maturation through co-aggregation and inflammatory modulation. These insights underscore the importance of developing targeted therapeutic strategies to address the unique challenges posed by different bacterial pathogens within biofilms.

SEM imaging provided additional insights into biofilm structure and treatment effects. While untreated biofilms showed a dense, interconnected fibrin matrix encapsulating bacterial cells, treated samples demonstrated partial interactions with EPS, particularly with LIS, OCT, and NaOCl. However, none of the solutions completely disrupted the biofilm matrix, which is reflected in the quantitative results. These observations align with previous studies showing that mature biofilms are challenging to penetrate due to their protective EPS layer and structural resilience [65]. This is due to the presence of extracellular polymeric substances (EPS), consisting of exopolysaccharides, (lipo)proteins, extracellular DNA (eDNA) and lipids. Oral antiseptic solutions are designed and approved to disrupt bacterial membranes and cell coats and to block prokaryotic membrane channels or enzyme chains and are not able to disrupt the “ingredients” of the EPS (polysaccharides, (lipo)proteins, eDNA, etc.), unlike enzymes. In addition, bacteria seem to migrate within the 3D biofilm structure, often relocating to deeper layers when challenged by antiseptics [65].

While the current ohpBIOM provides valuable insights into biofilm behavior under biologically relevant conditions, future improvements could enhance its clinical relevance. Optimization steps may include integrating flow dynamics and continuous nutritional support to better simulate the dynamic oral environment. Additionally, maintaining immune cell viability under prolonged anaerobic conditions remains a challenge and should be explored in future studies. Using patient-derived oral microbiomes could also improve the translational value of the model by reflecting individual microbial compositions. Finally, incorporating colony-forming unit (CFU) counts into the methodological toolkit would allow for the direct quantification of viable bacteria alongside molecular assessments, such as qPCR. This would strengthen the evaluation of antiseptic efficacy.

## 5. Conclusions

This translational study demonstrates the complexity of treating mature biofilms and highlights the importance of considering strain- and donor-specific variability in antimicrobial evaluations. The 3D human oral biofilm model used in this study incorporates donor-specific immune competence and provides a biologically relevant method of evaluating antimicrobial strategies. The key findings include:

Efficacy of antiseptic agents: Listerine exhibited the most consistent antibacterial effect with a near-significant reduction in bacterial load. Octenidine-based products (OCT and OCD) showed moderate activity, while chlorhexidine (CHX) was the least effective against mature biofilms, contrary to expectations.

Species-specific responses: Antiseptics demonstrated significantly varying efficacy across individual bacterial species, supporting the potential for targeted therapeutic strategies.

Donor variability: Treatment outcomes differed substantially between donors, emphasizing the influence of host-specific factors. On the one hand, this confirms that there is no ‘one-fits-all’ approach to antimicrobial therapy for infectious oral diseases; currently, it is more a case of choosing the best option available. On the other hand, it reinforces the fundamental concept of personalized treatment approaches.

Clinical implications and future research: The findings emphasize the importance of mechanical biofilm disruption during the treatment process of periodontitis and peri-implantitis. Future studies should focus on improving antiseptic penetration into oral biofilm matrices (EPS) and exploring combined therapeutic strategies to address the multifaceted resilience of mature biofilms.

## Data Availability

The data presented in this study are available upon request from the corresponding author. The data are not publicly available due to ownership rights held by the University Medical Center Hamburg-Eppendorf, 20246 Hamburg, Germany.

## Abbreviations

The following abbreviations are used in this manuscript:

BOP	Bleeding on probing
CaCl_2_	Calcium chloride
Cat. No.	Catalog number
CFU	Colony forming units
CHX	Chlorhexidine bis(D-gluconate)
CO_2_	Carbon dioxide
DNA	Deoxyribonucleic acid
DSM no.	Strain number assigned by the Deutsche Sammlung von Mikroorganismen und Zellkulturen (DSMZ), the German Collection of Microorganisms and Cell Cultures.
eDNA	Extracellular DNA
EFP	European Federation of Periodontology
EPS	Extracellular polymeric substance
FCS	Fetal calf serum
FFP	Fresh frozen plasma
GLM	Generalized linear model
HCl	Hydrochloric acid
LIS	Listerine^®^ Cool Mint
LRS	Leucocyte reduction system
MIC	Minimum inhibitory concentration
NaNO_2_	Sodium nitrite
NaOCl	Sodium hypochlorite
OCD	Octenident mouthwash
OCT	Octenisept
ohpBIOM	Human plasma oral biofilm model
OsO_4_	Osmium tetroxide
PBS	Phosphate-buffered saline
PD	Pocket probing depth
pH	Potential of hydrogen
qPCR	Quantitative polymerase chain reaction
RT	Room temperature
SAS	Statistical Analysis System
SEM	Scanning electron microscopy
SRP	Scaling and root planing
